# Focal solitary necrotic nodules in fatty liver: characteristics on conventional and contrast-enhanced ultrasonography

**DOI:** 10.1007/s40477-021-00634-3

**Published:** 2022-01-29

**Authors:** Yue-ling Peng, Li-ping Liu, Yan-jing Zhang, Jing-jing Liu, Xiao-Ling Yu

**Affiliations:** 1 Department of Nephrology, Shanxi People’s Hospital, Taiyuan, 030001 China; 2 Department of Ultrasound, First Hospital of Shanxi Medical University, Taiyuan, 030001 China; 3 Department of Interventional Ultrasound, Chinese People’s Liberation Army General Hospital, 28 Fuxing Rd, Beijing, 100853 China

**Keywords:** Solitary necrotic nodule, Fatty liver, Contrast agent, Ultrasonography

## Abstract

**Purpose:**

Focal lesions in fatty liver are difficult to diagnose using conventional ultrasonography (CVUS). The aim of this study was to investigate the characteristics of solitary necrotic nodules (SNNs) in fatty liver using CVUS and contrast-enhanced ultrasonography (CEUS) and to evaluate the diagnostic value of CEUS for SNNs in fatty liver.

**Methods:**

Fifteen SNNs in the fatty liver of fifteen patients were examined by both CVUS and CEUS. The contrast agent SonoVue was used for CEUS. The characterization and shape of these SNNs in the fatty liver were analyzed using CEUS.

**Results:**

CVUS revealed eight oval-shaped, six irregularly shaped, and one wedge-shaped SNN in the fatty liver. The six irregularly shaped lesions on CVUS were revealed to comprise four gourd-shaped, one serpiginous, and one 3-pin socket-shaped nodule on CEUS. One of these SNNs showed a subcapsular wedge shape, with peripheral and distinct internal septal hyperenhancement in the arterial phases that washed out in the portal phase; moreover, most areas of th lesion showed no internal enhancement in any of the three phases. Fourteen of the lesions were characterized as lacking internal enhancement, and 12 of them had mild–moderate peripheral thin enhancement in the arterial phases. Twelve of the 15 nodules could be considered for diagnosis as SNNs by CEUS, which was further proved by US-guided biopsy and histopathology. However none of them could be considered for diagnosis as SNNs by CVUS.

**Conclusions:**

CEUS is a valuable tool for visualizing the characteristics of SNNs in fatty liver to improve the differential diagnosis.

## Introduction

The incidence of fatty liver has increased over the past 2 decades; in fact, 25% of the world's population is currently thought to have nonalcoholic fatty liver disease (NAFLD) [1], and there is even a high prevalence in children [2, 3]. Ultrasonography is the first-line imaging modality for fatty liver and focal liver lesions. However, focal lesions in the fatty liver are difficult to diagnose using conventional ultrasonography (CVUS) [4, 5] and usually do not show the typical characteristics of malignant and benign lesions; for example, there may be no hypoechoic halo sign typical of malignant tumors and nonhyperechoic lesions of hemangiomas.

Solitary necrotic nodules (SNNs) of the liver was first described by Shepherd and Lee [6]. The etiology of SNNs of the liver is still unknown despite multiple hypotheses, including trauma [6], parasitic infestation [6, 7] and sclerotic hemangioma [6, 8], and allergic and defense reactions for a history of gastrointestinal cancer [9]. SNN of the liver is a rare and non-neoplastic lesion with a very challenging diagnosis [10, 11]. Studies on features on MRI, CT and contrast-enhanced ultrasonography (CEUS) of SNNs have been reported, but they are still frequently misinterpreted as malignant to surgical resection. Recently, it was reported that an SNN was misdiagnosed as metastasis by CT MRI and PET-CT and removed surgically. Finally, the SNN was proved by pathology to have a completely necrotic core and a fibrotic capsule containing infiltrating inflammatory cells [12]. Therefore, there is a need to continue further research on SNNs to improve the diagnostic awareness.

Low mechanical index (MI) real-time CEUS has greatly improved the differential diagnosis of focal liver lesions [5, 13–15]. Because of the increasing incidence of focal lesions in patients with fatty liver in recent years, some cases of SNNs in the liver have been collected since we have researched focal lesions in fatty liver for many years. We describe and analyze the characteristics of SNNs in fatty liver using CVUS and CEUS in this study to improve their diagnosis.

## Materials and methods

### Patients

During a 7-year period, a total of 15 patients with SNN lesions in the fatty liver were investigated using CEUS. There were 11 men and 4 women with a mean (± SD) age of 46.73 ± 7.32 years (34–58 years). There was no history of hepatitis B or HCV infection in any of these patients. In these patients, ultrasound examination found lesions, such as ten patients for health checkups; four patients for upper abdominal discomfort, including one with cholecystitis and gallbladder polyps; and one patient for postoperative follow-up of gastric cancer. One patient had alcoholism, 4 of whom had elevated aminotransferase.

The mean diameter of the lesions was 2.06 ± 0.89 cm (from 0.85 to 4.60 cm). The ultrasonographic diagnosis of fatty liver was established based on typical ultrasonographic findings: a markedly echogenic liver compared with the right kidney. In our study, SNNs and fatty livers were confirmed by ultrasound-guided biopsy. Written informed consent was provided by each patient to participate in research and to publish, and the study was approved by the ethics committee of Chinese People’s Liberation Army General Hospital and the first Hospital of Shanxi Medical University,

#### CVUS

Ten of the patients were imaged using a Sequoia 512 scanner (Siemens Medical Ultrasound, Mountain View, CA) equipped with contrast pulse sequencing (CPS) software, allowing for a real-time depiction of blood perfusion in the lesion at a low MI. A 4V1 vector transducer with a frequency range of 1–4 MHz was used. The remaining five patients were assessed using a Mylab_90 scanner (Esaote, SpA. Genoa, Italy), which has a CA431 vector transducer with a frequency range of 1–8 MHz equipped with contrast-tuned imaging (CnTI) software.

Baseline ultrasonography of the liver was performed to identify focal lesions, and the location, size, shape, margin, and posterior echo enhancement of the lesion were recorded. The pulse repetition frequency was set to the lowest value that was free of motion artifacts to maximize the color signal of slow-velocity blood flow.

#### CEUS

The contrast agent used in this study was SonoVue (Bracco, Milan, Italy), which was supplied as a lyophilized powder and reconstituted with 5 mL of saline to form a homogeneous microbubble suspension. All patients provided full informed consent prior to receiving the SonoVue injection. CEUS studies were performed after the administration of 2.4 mL of SonoVue into an antecubital vein in a bolus via a 20-gauge needle followed by a flush with 5 mL of normal saline.

CPS or CnTI was activated following the injection of the contrast agent. The MI value displayed on screen ranged from 0.13 to 0.17 for CPS and was 0.7 for CnTI. A dual-image real-time display was used to help locate the target lesion during the examination. Digital cine clips of typical CVUS images and all CEUS images were stored on the hard disks of the imaging system and transferred to personal computers for subsequent analysis.

### Imaging analysis

CVUS images and dynamic CEUS images were analyzed by two experienced radiologists (L.P.L. and X.L.Y.) in consensus. The CVUS and CEUS characteristics were recorded.

## Results

### Characteristics of the 15 SNNs in the fatty liver on CVUS

Twelve lesions were found in the right liver lobe, and three lesions were found in the left lobe. All SNNs in the fatty liver were hypoechoic lesions. Nine lesions had homogeneous internal echoes; All others had heterogeneous patterns. CVUS revealed eight oval-shaped, six irregularly shaped, and one wedge-shaped SNN. Eleven nodules had distinct margins, and the other four had indistinct margins. Four nodules had posterior echo enhancement. (Table 1).

**Table 1:** Conventional and Contrast-enhanced ultrasonography Characteristics of 15 SNNs

No.	Sex/age	Location	Size (cm)	6794525082500CVUS				10223525781000CEUS	
				Lesion margin	Internal echo	Posterior echo enhancement	Shape	Lesion margin	Shape
1	F/58	Right anterior superior segment	2.0	Indistinct	Homo	Absent	Irregular	Distinct	serpiginous
2	M/45	Right posterior segment	2.5	Distinct	Homo	Absent	Irregular	Distinct	3-pin socket-shaped
3	F/49	Right posterior segment	1.3	Distinct	Homo	Absent	Oval	Distinct	Oval
4	F/50	Right posterior segment	2.3	Distinct	Homo	Absent	Irregular	Distinct	Gourd-shaped
5	F/55	Right anterior inferior segment	1.3	Distinct	Homo	Absent	Oval	Distinct	Oval
6	M/38	Left lateral segment	0.85	Distinct	Homo	Absent	Oval	Distinct	Oval
7	M/43	Right anterior superior segment	1.8	Distinct	Heter	Absent	Oval	Distinct	Oval
8	M/44	Right anterior segment	1.2	Distinct	Homo	Absent	Oval	Distinct	Oval
9	M/55	Right posterior segment	2.45	Distinct	Homo	Absent	Irregular	Distinct	Gourd-shaped
10	M/34	Right anterior inferior segment	4.6	Distinct	Heter	Slight enhancing	Wedge	Distinct	Wedge
11	M/45	Right anterior inferior segment	2	Distinct	Heter	Absent	Oval	Distinct	Oval
12	M/56	Right posterior segment	1.1	Indistinct	Heter	Absent	Irregular	Distinct	Gourd-shaped
13	M/45	Left lateral segment	1.8	Distinct	Heter	Slight enhancing	Oval	Distinct	Oval
14	M/48	Right anterior inferior segment	2.6	Indistinct	Heter	Slight enhancing	Irregular	Distinct	Gourd-shaped
15	M/36	Left lateral segment	1.76	Indistinct	Homo	Slight enhancing	Oval	Distinct	Oval

Only one wedge-shaped lesion located subcapsularly exhibited peripheral blood vessels on color Doppler flow imaging (CDFI), and the remaining fourteen nodules showed no color signal.

Using CVUS, only four lesions were determined to be benign; however, none of them could be diagnosed as SNNs.

### Characteristics of SNNs in fatty liver on CEUS

**Fig. 1:**
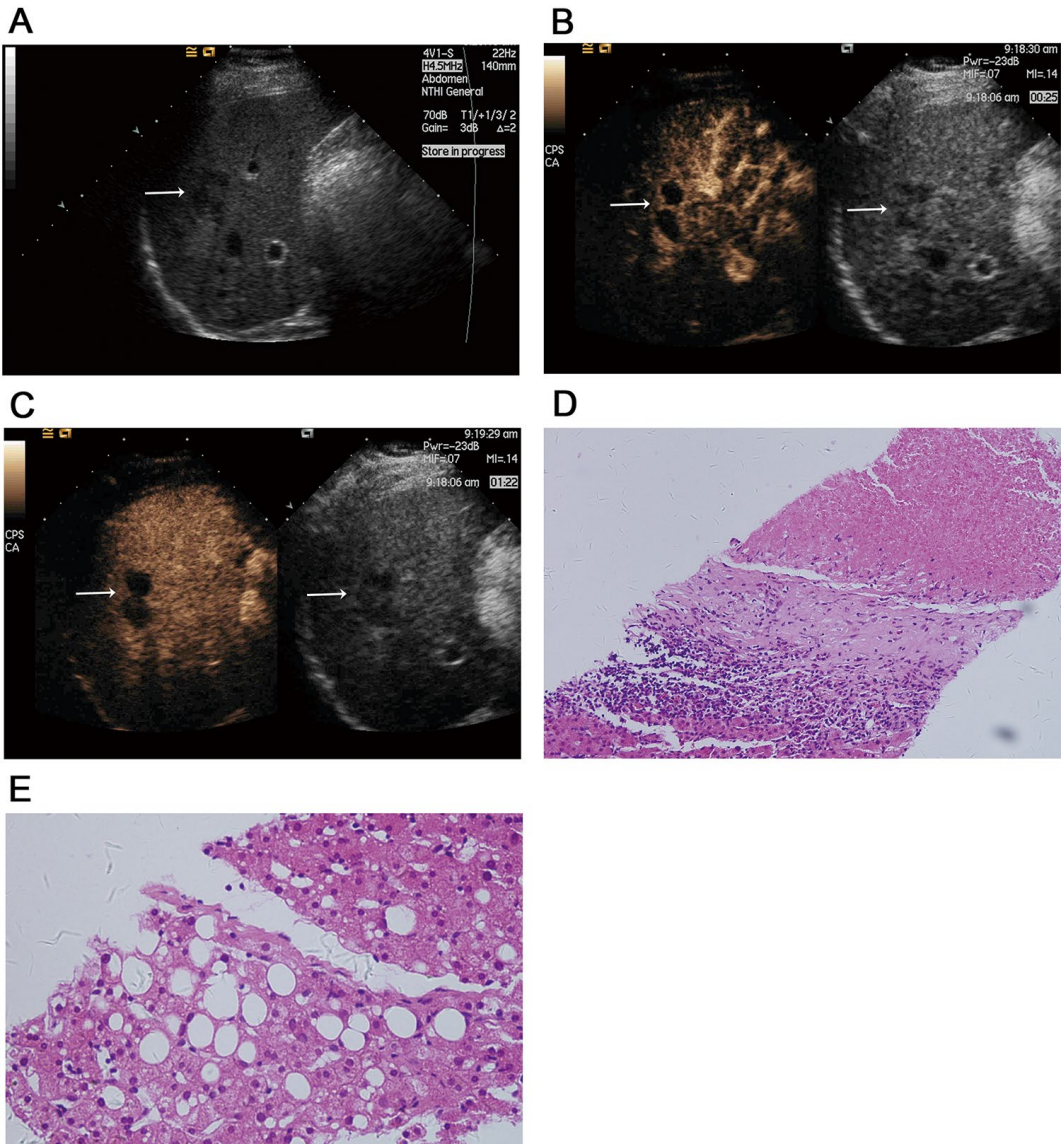
Focal hypoechoic lesion (SNN) in a 48-year-old man with fatty liver. **A** Sonogram showing the focal irregular hypoechoic lesion (arrow) in the right lobe of the fatty liver; **B** Contrast-enhanced image acquired with CPS showing mild–moderate peripheral enhancement in a gourd-shaped lesion in the arterial phase (left arrow), with no enhancement within the lesion, corresponding to the hypoechoic lesion (right arrow) seen in the fatty liver on the baseline sonogram; **C** No enhancement was seen within the lesion in the portal phase, corresponding to the hypoechoic lesion (right arrow) seen in the fatty liver on the baseline sonogram; **D** Histology of the liver biopsy specimen showing central coagulation necrosis surrounded by fibrous tissue with infiltrating inflammatory cells (hematoxylin–eosin, original magnification × 200); **E** Histology of biopsy specimen of the surrounding hepatic parenchyma showing steatosis and balloon cells (hematoxylin–eosin, original magnification × 200)

**Fig. 2:**
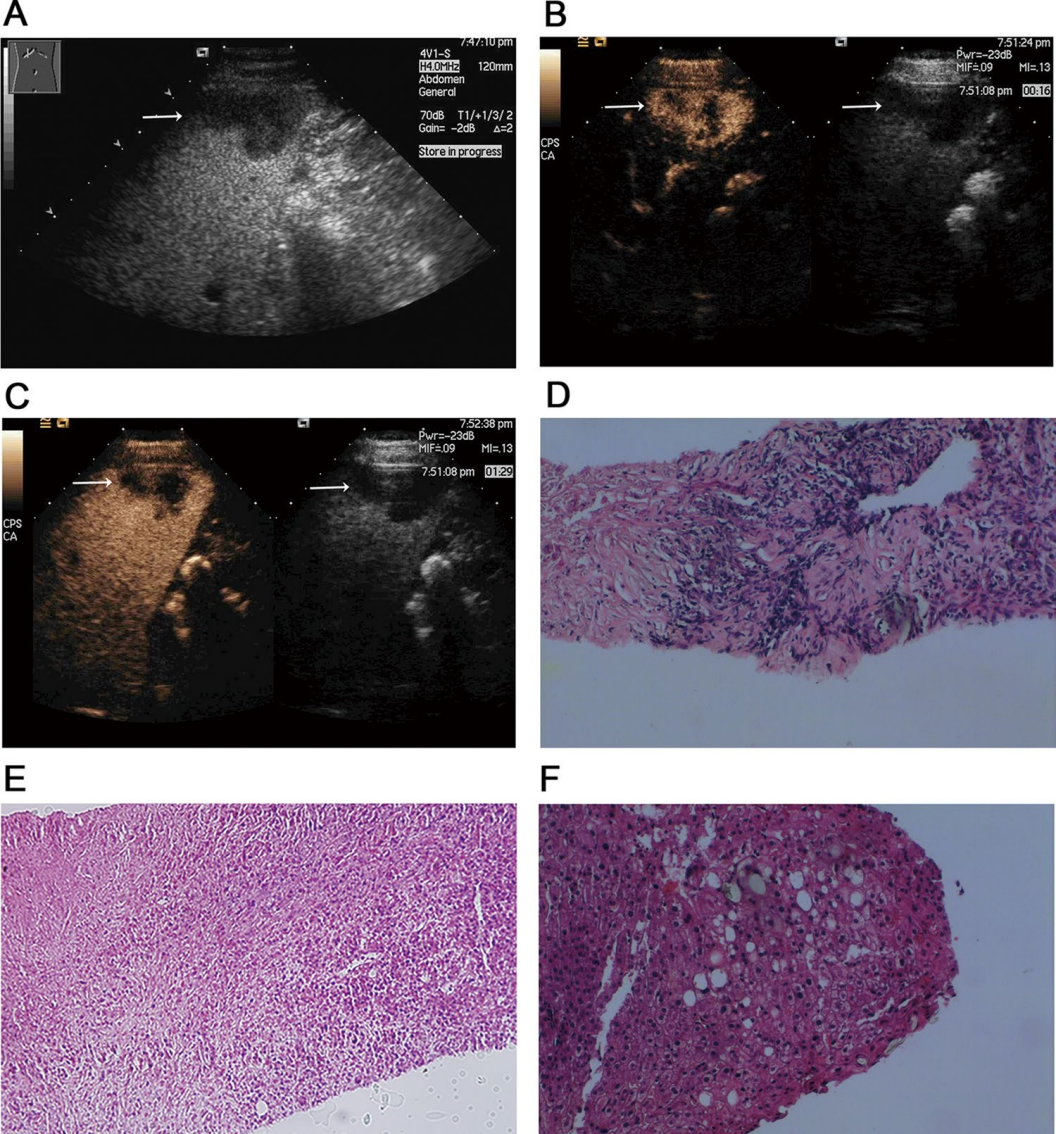
Focal hypoechoic lesion (SNN) in a 34-year-old man with fatty liver. **A** Sonogram showing the focal wedge-shaped hypoechoic lesion (arrow) in the subcapsular region of the fatty liver; **B** contrast-enhanced image acquired with CPS showing peripheral and septal enhancement, with no internal enhancement during the arterial phase (left arrow), corresponding to the hypoechoic lesion (right arrow) seen in the fatty liver on the baseline sonogram; **C** contrast-enhanced image acquired with CPS showing a likely SNN with wash out in the portal phase, corresponding to the hypoechoic lesion (right arrows) seen in the fatty liver on the baseline sonogram; **D** histology of biopsy specimen showing coagulation necrosis (hematoxylin–eosin, original magnification × 200); **E** histology of biopsy specimens showing fibrous tissue with infiltrating inflammatory cells (hematoxylin–eosin, original magnification × 200); **F** biopsy specimen confirming the surrounding hepatic parenchyma as fatty liver with steatosis and balloon cells (hematoxylin–eosin, original magnification × 200)

**Fig. 3:**
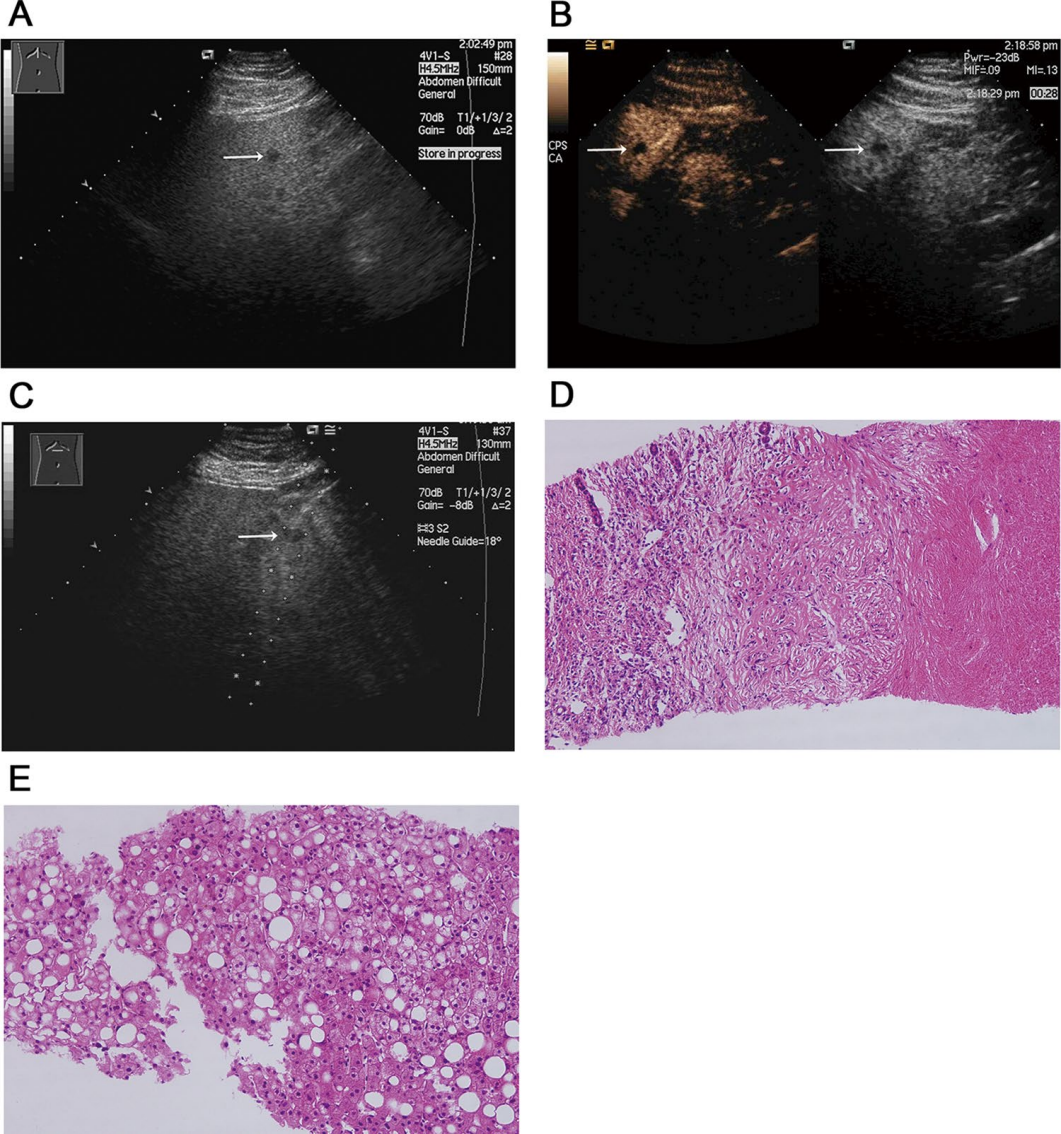
Focal hypoechoic lesion (SNN) in a 37-year-old man with fatty liver. **A **Sonogram showing the focal hypoechoic lesion (arrow) in the left lobe of the fatty liver; **B** contrast-enhanced image acquired with CPS showing no enhancement in the lesion (left arrow) through all three phases, corresponding to the hypoechoic lesion (right arrow) seen in the fatty liver on CVUS. **C** US-guided biopsy of the lesion; **D** histology of biopsy specimen showing necrosis surrounded with a fibrotic capsule and infiltration of inflammatory cells (hematoxylin–eosin, original magnification × 200); **E** histology of biopsy specimen showed the surrounding hepatic parenchyma as fatty liver with steatosis and balloon cells (hematoxylin–eosin, original magnification × 200)

On CEUS, eight of the SNNs were oval shaped, and one was wedge shaped. The six irregularly shaped lesions on CEUS were revealed to comprise four gourd-shaped, one serpiginous, and one 3-pin socket-shaped nodule using CEUS. All lesion margins were distinct on CEUS. (Figs. 1, 2, 3, Table 1).

Twelve of the SNNs showed mild-moderate peripheral thin enhancement in the arterial phase, which became identical to the signal in the liver parenchyma in the portal and delayed phases with no enhancement in the interior of the lesion (Fig. 1). One wedge-shaped SNN was characterized by peripheral and distinct internal septal hyperenhancement in the arterial phases, and hypoenhancement in the portal and delayed phases (Fig. 2), moreover, most areas of this lesion showed no internal enhancement in any of the three phases; otherwise, the other nodules showed no interior enhancement. The other two lesions had no enhancement in any of the three phases.

Twelve lesions could be considered for the diagnosis of SNNs using CEUS, which was further proven by US-guided biopsy and histopathology. The smallest lesion could still not be determined to be a cyst or SNN due to the lack of interior enhancement in all three phases with no thin peripheral enhancement in the arterial phase; subsequently, this lesion was diagnosed as an SNN by US-guided biopsy and histopathology (Fig. 3). One wedge-shaped lesion was thought to be an inflamed and possibly infected lesion but was proven by US-guided biopsy and histopathology later to be an SNN (Fig. 2).

## Discussion

SNN of the liver is usually benign. The histopathology of SNN demonstrates central coagulation necrosis with a peripheral fibrous capsule and infiltration of inflammatory cells. Most patients generally had no clinical symptoms, which were accidentally detected during ultrasound examination. Because of inadequate knowledge of SNN of the liver, it is often interpreted as malignant, leading to surgery. Chen et al. [16] reported one SNN of the liver that was indeterminate on both abdominal CVUS and MRI, while F-18 fluorodeoxyglucose positron emission tomography/computed tomography (F-18 FDG PET/CT) showed a solitary hypermetabolic mass in the liver; therefore, malignancy was suspected, and postoperative pathology showed an SNN with larval infestation. De Luca et al. [17] reported one SNN of the liver with compression of the celiac axis (Dunbar syndrome); after surgery, the pathological diagnosis of this focal lesion was SNN due to ischemic injury.

In our study, two SNNs with likely hypoechoic halos were difficult to distinguish from hepatic metastasis using CVUS; however, they were diagnosed using CEUS. In our study, all SNNs were hypoechoic against a fatty liver background on CVUS, four lesions had posterior echo enhancement, and four were characterized by indistinct margins. Using CVUS, only four lesions were determined to be benign, but none of them could be diagnosed as SNNs. Twelve of fifteen lesions were correctly considered for the diagnosis of SNNs using CEUS, which was further proven by US-guided biopsy and histopathology.

SNNs can often be easily misdiagnosed as malignant lesions of the liver. Yoon et al. [18] reported that two SNNs of the liver mimicked metastasis on radiologic images. One of the lesions in arterial- and portal-phase spiral CT showed focal wall thickening with enhancement in the fundus of the gallbladder and a 1.0 cm ovoid hypoattenuating nodule with peripheral rim enhancement in the right lobe of the liver, which suggested gallbladder cancer with hepatic metastasis. After surgery, histopathology revealed that the gallbladder lesion was an adenocarcinoma; however, the hepatic lesions were SNNs composed of a central necrotic core and a peripheral fibrotic capsule with inflammatory cells.

Delis et al. [19] reported a 7 cm hyperechoic lesion in the right liver lobe using abdominal US. Contrast-enhanced CT demonstrated encapsulation with peripheral enhancement, a necrotic core and contrast washout in the venous phase, which led to a suspicion of adenoma or hepatocellular carcinoma (HCC), although fever and leukocytosis suggested a possible infectious process. The patient was treated with intravenous antibiotics for 7 days until the fever subsided and the complete white cell count normalized. Due to the high suspicion for a malignancy, postoperative pathology was performed and indicated a hemorrhagic infarct and extensive hepatocellular necrosis due to sinusoidal portal vein thrombosis.

Wang et al. [20] reported 17 SNNs in the liver, of which eight were oval, and the others were lobulated. However, the actual shapes of some SNNs in the fatty liver cannot be easily visualized on CVUS. In our study, six lesions were characterized as irregularly shaped on CVUS. Using CEUS, we found that four were gourd shaped (Fig. 1), one was serpiginous shaped, and one was 3-pin socket-shaped. CEUS distinctly revealed the shape and margin of SNNs in the fatty liver, and some of these lesions exhibited typical SNN morphology, such as a gourd shape. Pananwala et al. [7] reported three SNNs of the liver that had a serpiginous shape with areas of necrosis.

Chunyu Lu et al. [21] reported that 13 SNNs (13/24, 54.1%) presented with peripheral thin rim-like enhancement in the arterial phase, isoenhancement in the portal phase and delayed phase with no enhancement in the interior of the lesions during three phases. Twelve of the SNNs in our study showed mild–moderate thin peripheral enhancement in the arterial phase, which is similar to the rim enhancement reported by Kim for SNNs of the liver in the arterial phase of MRI [22]. Francica et al. [23] reported that 10 SNNs (10/24, 41.6%) occurred thin, uniform, hyperenhancing ring in the early arterial on CEUS, but 2 nodules (2/7, 28.6%) showed late rim enhancement in contrasted-enhanced MRI. Fang et al. [24] reported SNNs in the liver with a prolonged delayed MRI time; when it was up to 1 h, all lesions represented moderate/marked peripheral enhancement with internal hypointensity. There is a difference between MRI contrast agents and ultrasound contrast agents. The nonspecific contrast agent in the extracellular space used in MRI, such as the delayed enhancement of the thin ring edge, may be the result of the slow diffusion, penetration and clearance of the contrast agent in the wide extracellular space. SonoVue is a blood pool contrast agent, that will not diffuse and penetrate the cell space and is usually excreted with breathing for 6 min. The pathological basis of peripheral thin enhancement in the arterial phase on CEUS is peripheral fibrous tissue with inflammatory cell infiltration.

The smallest lesion in our study did not demonstrate ring-like enhancement in the arterial phases (Fig. 3); furthermore, after US-guided biopsy, histopathology determined this lesion to be an SNN, which was missed by CT because the lesion was too small, measuring at just 0.85 cm. It should be noted that the pathological diagnosis of surgically resected specimens is not difficult, but coagulation necrosis, fibrous bands and basically normal hepatocytes must be seen under the microscope in the diagnosis of ultrasound-guided biopsy specimens [25].

In our study, one wedge-shaped lesion demonstrated a blood flow signal in the periphery, and CEUS revealed peripheral and septal enhancement in the arterial phase, which likely became washed out in the portal phase (Fig. 2), and most of the center showed no enhancement. This patient had a history of injury that may be consistent with the SNN hypothesis based on trauma [6]. The following year, the patient underwent surgery at his resident city’s hospital; furthermore, postoperative pathology showed the same result as our biopsy and histopathology analysis.

For patients receiving interventional therapy for HCC or hepatic metastasis, CEUS revealed no enhancement with coagulation necrosis resembling SNN, so inquiring about the medical history of each patient is important for diagnosis. For some ovarian SNNs, the mild–moderate thin ring-like enhancement in the arterial phase becomes isoenhancement in the portal or late phases with no internal enhancement in any of the three phases, which requires differential diagnosis from metastatic cancer. For some hepatic metastatic lesions, ring-like or peripheral enhancement is observed during the arterial phase but it is inhomogeneous and thicker peripheral enhancement, further changes to hypoenhancement in the portal and late phases. Deniz et al. [26] reported that SNNs in the liver are not always benign, which is important to identify minute foci of metastatic carcinoma. Thus, we should combine the patient's medical history, tumor marker test, and further ultrasound-guided puncture biopsy.

On follow-up with our patients, two SNNs in the liver had regressed, and another two of SNNs had calcified. Choi et al. [27] reported that a hepatic mass (diameter 2 cm) was diagnosed as an SNN by liver biopsy, and follow-up for 7 months revealed that the SNN had spontaneously regressed. Thus, conservative treatment and follow-up should be a good management option for SNNs.

## Conclusion

CEUS is a superior method for detecting SNNs in fatty liver compared to CVUS. Using CEUS, the diagnosis of SNNs in the fatty liver can be made according to lesion shape and enhancement characteristics, and proven by US-guided biopsy and histopathology to avoid unnecessary surgery. Overall, CEUS is a valuable tool for the diagnosis of SNNs in fatty liver.
